# Blood Levels of Glutamate and Glutamine in Recent Onset and Chronic Schizophrenia

**DOI:** 10.3389/fpsyt.2018.00713

**Published:** 2018-12-19

**Authors:** Caroline Madeira, Flavio V. Alheira, Marilia A. Calcia, Thuany C. S. Silva, Filippe M. Tannos, Charles Vargas-Lopes, Melissa Fisher, Nelson Goldenstein, Marco Antonio Brasil, Sophia Vinogradov, Sergio T. Ferreira, Rogerio Panizzutti

**Affiliations:** ^1^Instituto de Ciências Biomédicas, Universidade Federal do Rio de Janeiro, Rio de Janeiro, Brazil; ^2^Serviço de Psiquiatria e Psicologia Médica, Hospital Universitário Clementino Fraga Filho, Universidade Federal do Rio de Janeiro, Rio de Janeiro, Brazil; ^3^Department of Psychiatry, School of Medicine, University of California, San Francisco, San Francisco, CA, United States; ^4^Department of Psychiatry, University of Minnesota, Minneapolis, MN, United States; ^5^Instituto de Bioquímica Médica Leopoldo de Meis, Universidade Federal do Rio de Janeiro, Rio de Janeiro, Brazil; ^6^Instituto de Biofísica Carlos Chagas Filho, Universidade Federal do Rio de Janeiro, Rio de Janeiro, Brazil

**Keywords:** recent onset schizophrenia, chronic schizophrenia, glutamate, glutamine, blood

## Abstract

Converging evidence indicates that dysfunctions in glutamatergic neurotransmission and in the glutamate-glutamine cycle play a role in the pathophysiology of schizophrenia. Here, we investigated glutamate and glutamine levels in the blood of patients with recent onset schizophrenia or chronic schizophrenia compared to healthy controls. Compared with healthy controls, patients with recent onset schizophrenia showed increased glutamine/glutamate ratio, while patients with chronic schizophrenia showed decreased glutamine/glutamate ratio. Results indicate that circulating glutamate and glutamine levels exhibit a dual behavior in schizophrenia, with an increase of glutamine/glutamate ratio at the onset of schizophrenia followed by a decrease with progression of the disorder. Further studies are warranted to elucidate the mechanisms and consequences of changes in circulating glutamate and glutamine in schizophrenia.

## Introduction

Schizophrenia is a heterogeneous disorder characterized by a wide range of symptoms, including positive (hallucinations, delusions, disorganized thinking) and negative symptoms (affective flattening, alogia, apathy), and cognitive impairments (in memory and executive functions) (1, 2). Considerable evidence indicates that dysfunctional glutamatergic neurotransmission is involved in the pathophysiology of schizophrenia (3, 4). Among different glutamate receptor subtypes, hypofunction of N-methyl-D-aspartate (NMDA) receptors has been particularly implicated in schizophrenia. This comes from observations that administration of the NMDA receptor antagonists, phencyclidine (PCP) and ketamine, induces schizophrenia-like symptomatology in healthy individuals and exacerbates symptoms in patients with schizophrenia (5–7). Additionally, dysfunctions in metabotropic glutamate receptors (mGluRs), which can modulate NMDA receptor function, have also been implicated in schizophrenia (8, 9). Increased levels of mGluR1protein and mRNA have been reported in schizophrenia patients (10, 11). mGluR2/3 agonists improved both positive and negative symptoms of schizophrenia patients (12) and reduced cognitive deficits induced by ketamine in healthy volunteers (13). Finally, a polymorphism in the mGluR7 gene was associated with schizophrenia (14).

Glutamatergic neurotransmission is initiated by the release of glutamate into the synaptic cleft, where it binds to and activates glutamate receptors at the postsynaptic terminal membrane. Glutamate is subsequently removed from the synaptic cleft by astrocytes and converted to glutamine by glutamine synthetase (15). Glutamine can then be transported into the presynaptic neuron and reconverted to glutamate by phosphate-activated glutaminase (16, 17). Abnormalities in the glutamate-glutamine cycle have been associated with schizophrenia and may contribute to the dysfunction in glutamatergic neurotransmission (18, 19). Evidence indicates that glutamine synthetase protein levels are decreased in schizophrenia (19, 20). On the other hand, other studies found increased expression and enzymatic activity of the phosphate-activated glutaminase in schizophrenia (18, 21).

Changes in glutamate levels in the central nervous system (CNS) may reflect changes in blood levels and vice-versa, since a positive correlation has been reported between glutamate levels in the blood and in the CNS (22, 23). Altered glutamate and glutamine levels in the blood have been reported in schizophrenia, but results are not consistent. For example, previous studies have found increased blood levels of glutamate in patients with chronic schizophrenia compared with healthy controls (24, 25), but no difference was observed in acute schizophrenia (26). Interestingly, one study reported decreased blood glutamate levels in first episode psychosis (27). Blood glutamine levels were reported to be decreased in patients with schizophrenia (28), although another study found no difference in blood glutamine levels of individuals with schizophrenia compared with healthy controls (26).

The above findings indicate that changes in peripheral glutamate and glutamine may occur in schizophrenia, but the direction of change appears to depend on the duration of the disorder. We now report measurements of glutamate and glutamine levels in the blood of patients with recent onset schizophrenia or chronic schizophrenia compared to healthy controls.

## Materials and Methods

### Participants

Thirty-two patients with recent onset (within 5 years of illness onset) and 56 patients with chronic schizophrenia were recruited from community mental health centers and outpatient clinics in the Bay Area of San Francisco, USA, to participate in randomized controlled trials of computerized cognitive training (ClinicalTrials.gov NCT00312962 and NCT00694889) (29–31). Blood collection was performed prior to the beginning of cognitive training. Fifty-three healthy control subjects were recruited from the same local community in the USA. In Brazil, 67 patients with chronic schizophrenia were recruited at the Institute of Psychiatry of the Federal University of Rio de Janeiro (IPUB-UFRJ) and the Psychiatry Center of Rio de Janeiro (CPRJ). Seventy-five healthy control subjects were recruited from the same local community in Brazil. Ethical approval for this study was obtained from the Committee for Ethics in Human Research from each participant institution. All subjects or their legal guardian provided written informed consent for study participation.

Patients with schizophrenia were diagnosed using the Structured Clinical Interview (SCID-I) for DSM-IV and evaluated using the Brief Psychiatry Rating Scale (BPRS). We used the BPRS because it is briefer and less time consuming than the Positive and Negative Syndrome Scale (PANSS), and there is a strong linear association between the two scales (32). Exclusion criteria were comorbid psychiatric diagnosis, substance abuse, intellectual disability, renal disease, epilepsy, and history of trauma or tumor in the central nervous system. Healthy controls had good general physical health and no Axis I or Axis II psychiatric disorder (SCID-NP), and no current or previous use of psychotropic medication.

### Determination of Glutamate and Glutamine Levels

Available samples comprised serum from the two clinical trial cohorts in the USA and plasma from the cross-sectional study in Brazil. Importantly, we note that a recent meta-analysis showed that altered levels of glutamate are found in both plasma and serum of patients with schizophrenia (33). Blood samples from patients or healthy controls were drawn before lunch, around noon, and stored at −80°C until analysis. To extract free amino acids, trichloroacetic acid (TCA; 5% final concentration) was added to each sample, followed by centrifugation for 5 min. at 14,500 rpm at room temperature, separation of the supernatant and removal of TCA by extraction with saturated ether. Derivatization was performed with o-phtalaldehyde and N-tert-butyloxycarbonyl-L-cysteine (34). Separation and detection of derivatized amino acids was performed by high-performance liquid chromatography (34, 35) with a linear gradient of acetonitrile using a C18 reverse phase column. The investigators (CM and CVL) were blind to the group identity of the samples.

### Statistical Analysis

Data are presented as means ± SD (standard deviation). The distributions of glutamate and glutamine concentrations were evaluated for missing values and normalcy. We adjusted outliers by winsorization (36) replacing them with the next value plus or minus 10% (depending on whether the outlier is positive or negative). For glutamate, two outliers were adjusted in the healthy control group and two outliers were adjusted in the recent onset schizophrenia group. For the glutamine/glutamate ratio, two outliers were adjusted in the healthy control group and two outliers were adjusted in the recent onset schizophrenia group. In the chronic schizophrenia cohort from the USA, for glutamate we adjusted one outlier in the healthy control group and two outliers in the chronic schizophrenia group. For glutamine, we adjusted three outliers in the chronic schizophrenia group. For the glutamine/glutamate ratio, we adjusted one outlier in the healthy control group and three outliers in the chronic schizophrenia group. In the chronic schizophrenia cohort from Brazil, for glutamate we adjusted three outliers in the healthy control group and four outliers in the chronic schizophrenia group. For glutamine, we adjusted one outlier in the healthy control group and two outliers in the chronic schizophrenia group. For the glutamine/glutamate ratio, we adjusted three outliers in the healthy control group and three outliers in the chronic schizophrenia group. Statistical significances of differences between groups were determined by unpaired *t*-test with Welch's correction. Differences between groups for gender and smoking status were analyzed using chi-square. Differences in glutamate or glutamine levels between healthy controls and patients with schizophrenia (recent onset or chronic) groups were tested using ANCOVA with age, sex or smoking status as covariates as appropriate.

## Results

### Blood Glutamate and Glutamine Levels in Patients With Recent Onset Schizophrenia

We first asked whether circulating levels of glutamate and glutamine were altered in a cohort of 32 patients with recent onset schizophrenia (5 or less years of disorder) compared with 38 healthy controls. The mean age of patients with recent onset schizophrenia was 2 years older than the mean age of the healthy controls and this difference was significant at group level (*t* = 2.5, *p* = 0.01) (Table 1). However, no significant correlations were observed between age and blood levels of glutamate or glutamine in either healthy controls or recent onset schizophrenia groups (Figure S1). Sex distribution was different between groups, with the schizophrenia group including more men (*X*^2^ = 8.07, *p* = 0.005). No significant difference in glutamine levels was observed between male and female healthy controls (Figure S2). In contrast, glutamate levels were significantly higher in males than in female patients with recent onset schizophrenia (151.7 ± 15.0 vs. 83.4 ± 16.8 μmol/L, respectively; *t* = 2.28; *p* = 0.03; Figure S2).

**Table 1:** Characteristics of study subjects with recent onset or chronic schizophrenia from the USA cohort.

	**Healthy controls**	**Recent onset schizophrenia**	***p***	**Healthy controls**	**Chronic schizophrenia**	***p***
Sex, M/F	17/21	25/7	0.005	10/5	40/16	0.72
Age, years (range)	18.8 ± 3.8 (12–25)	21.0 ± 3.7 (15–29)	0.015	44.9 ± 11.2 (26–57)	45.9 ± 8.3 (26–59)	0.75
Illness duration, years (range)	N/A	1.82 ± 1.68 (0.08–5)	N/A	N/A	23.6 ± 10.21 (6–41)	N/A
CPZ equivalents (mg/day) (range)	N/A	243.3 ± 133.9 (25.1–562.9)	N/A	N/A	402.7 ± 330.2 (0–1918.0)	N/A
Antipsychotic in use (typical/atypical/combination/no medication)	N/A	1/24/1/5	N/A	N/A	7/42/3/2	N/A
Smoker/ Non-smoker	1/36	20/8	0.003	3/12	31/25	0.015
BPRS total score (range)	N/A	42.5 ± 9.2 (26–62)	N/A	N/A	35.2 ± 9.2 (18–56)	N/A
BPRS negative score (range)	N/A	16.5 ± 6.4 (8–36)	N/A	N/A	17.6 ± 6.3 (9–38)	N/A
BPRS positive score (range)	N/A	13.7 ± 4.8 (7–22)	N/A	N/A	18.4 ± 5.5 (8–29)	N/A

*Comparisons between the two groups were performed using χ^2^ test for sex and smoking status, and t-test for age. Values are shown as means ± standard deviation (range). BPRS, Brief Psychiatric Rating Scale; CPZ, chlorpromazine; N/A, not applicable. Typical antipsychotics included flufenazine, clorpromazine, amoxapine, haloperidol, perphenazine, thioridazine, and thiothixene. Atypical antipsychotics included clozapine, olanzapine, quetiapine, risperidone, ziprasidone, and aripiprazol*.

The proportion between smokers and non-smokers was also different between the two groups (*X*^2^ = 8.94, *p* = 0.003). The group of patients with recent onset schizophrenia included more smokers than the healthy control group (28.6 vs. 2.7% smokers, respectively). We therefore introduced age, sex and smoking status as covariates in the analysis and found that blood glutamate and glutamine levels were ~30% lower in patients with recent onset schizophrenia compared to healthy controls (Table 2). Further, the glutamine/glutamate ratio was significantly higher in patients with recent onset schizophrenia than in healthy controls (Table 2).

**Table 2:** Blood levels of glutamate and glutamine in recent onset schizophrenia (USA cohort) adjusted for age, sex, and smoking status as covariates.

			**95% C.I**.	
**Amino acids**	**Group**	**Mean**	**Lower bound**	**Upper bound**	**Statistics**
Glutamate, μmol/L	Healthy controls	201.5	177.3	225.8	*F* = 12.6, *p* = 0.001
	Recent onset schizophrenia	131.2	102.9	159.6
Glutamine, μmol/L	Healthy controls	505.3	459.0	551.5	*F* = 12.8, *p* = 0.001
	Recent onset schizophrenia	370.4	316.3	424.5
Glutamine/ glutamate ratio	Healthy controls	2.73	1.99	3.46	*F* = 4.47, *p* = 0.04
	Recent onset schizophrenia	3.99	3.14	4.85

*C.I., confidence interval*.

In patients with recent onset schizophrenia, glutamate and glutamine levels were not significantly correlated to total BPRS score (*r* = 0.22, *p* = 0.22 for glutamate; *r* = −0.33, *p* = 0.06 for glutamine), to the BPRS score for negative symptoms (*r* = 0.19, *p* = 0.28 for glutamate; *r* = −0.21, *p* = 0.24 for glutamine), or to the BPRS score for positive symptoms (*r* = 0.16, *p* = 0.39 for glutamate; *r* = −0.27, *p* = 0.13 for glutamine). Moreover, no significant correlation was observed between use of antipsychotic medications (expressed as chlorpromazine equivalents) and glutamate or glutamine levels (*r* = −0.09, *p* = 0.66 for glutamate; *r* = 0.17, *p* = 0.43, for glutamine; Figure S3).

### Blood Glutamate and Glutamine Levels in Patients With Chronic Schizophrenia

Next, we asked whether patients with chronic schizophrenia showed altered glutamate and glutamine levels compared to healthy controls. Patients with chronic schizophrenia and healthy controls had similar age and sex distributions, but patients with schizophrenia were more likely to smoke (*X*^2^ = 5.93, *p* = 0.015) (Table 1). The smoking status was thus included as a covariate in the analysis (Table 3). Patients with chronic schizophrenia showed a trend of increase in blood glutamate levels compared to healthy controls (*F* = 3.50; *p* = 0.066). On the other hand, glutamine levels were significantly lower in patients with chronic schizophrenia than in healthy controls (*F* = 19.82; *p* = 0.0001). As a result, the glutamine/glutamate ratio was lower in patients with chronic schizophrenia than in healthy controls (*F* = 32.05, *p* = 0.0001).

**Table 3:** Blood levels of glutamate and glutamine in chronic schizophrenia (USA cohort) using smoking status as covariate.

			**95% C.I**.	
**Amino acids**	**Group**	**Mean**	**Lower bound**	**Upper bound**	**Statistics**
Glutamate, μmol/L	Healthy controls	180.3	101.1	259.5	*F =* 3.5, *p =* 0.066
	Chronic schizophrenia	254.6	221.4	287.8
Glutamine, μmol/L	Healthy controls	472.8	377.1	568.6	*F =* 19.8, *p =* 0.0001
	Chronic schizophrenia	259.1	219.0	299.3
Glutamine/ glutamate ratio	Healthy controls	4.13	3.25	5.02	*F =* 32.0, *p =* 0.0001
	Chronic schizophrenia	1.62	1.25	1.99

*C.I., confidence interval*.

No significant correlation was observed between age and levels of glutamate or glutamine in healthy controls (Figure S4). In contrast, age was significantly correlated with glutamate (*r* = 0.30, *p* = 0.02) and glutamine levels (*r* = −0.27, *p* = 0.04) in patients with chronic schizophrenia (Figure S4).

**Figure 1:**
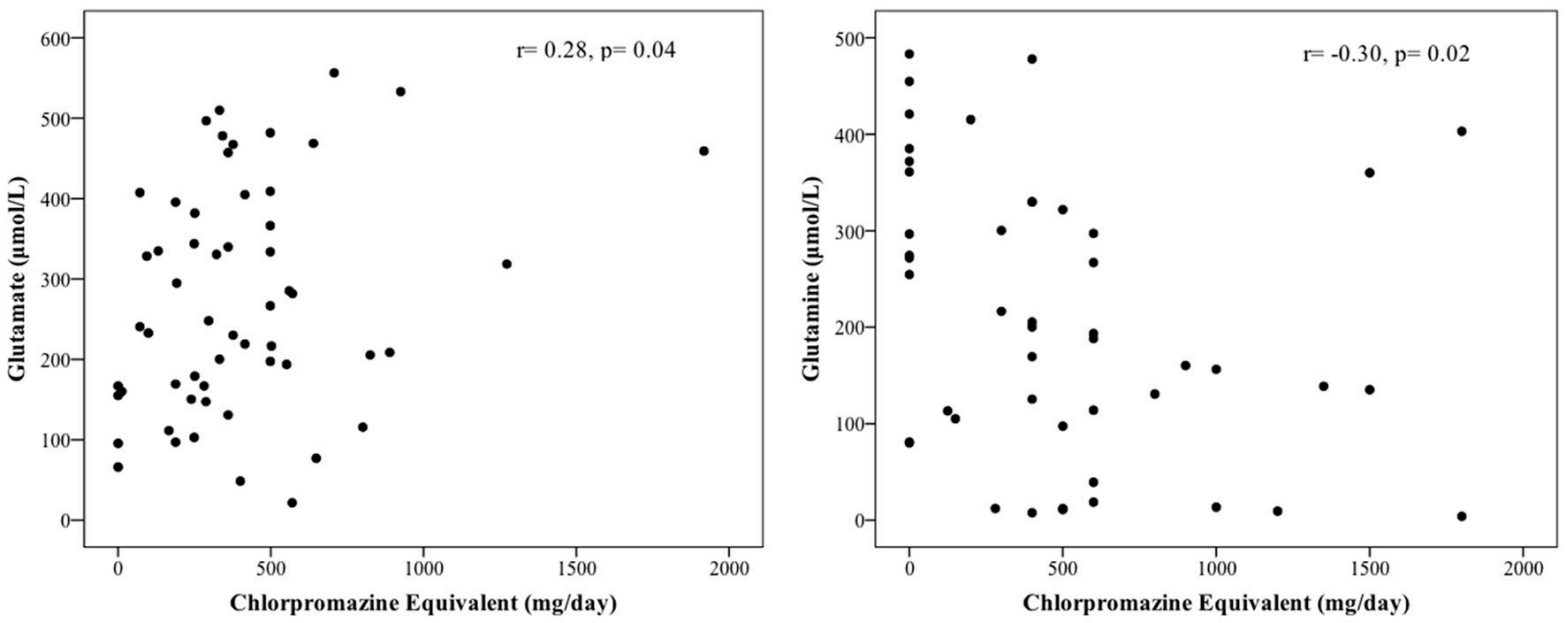
Correlation between blood levels of glutamate and glutamine and the use of antipsychotic medication in chronic schizophrenia (USA cohort).

We evaluated the correlation between blood amino acid levels and the current use of medications and found that glutamate and glutamine levels were significantly and negatively correlated to the daily use of antipsychotic medications (measured in chlorpromazine equivalents) (*r* = 0.28, *p* = 0.04 for glutamate; *r* = −0.30, *p* = 0.02, for glutamine; Figure 1). We further evaluated the correlation between blood glutamate and glutamine levels and the severity of symptoms in patients with chronic schizophrenia. Glutamate and glutamine levels were correlated to total BPRS score (*r* = −0.35, *p* = 0.01 for glutamate; *r* = 0.26, *p* = 0.05 for glutamine), but not to the BPRS score for positive symptoms (*r* = −0.25, *p* = 0.06 for glutamate; *r* = 0.05, *p* = 0.72 for glutamine) or to the BPRS score for negative symptoms (*r* = −0.07, *p* = 0.63 for glutamate; *r* = 0.1, *p* = 0.48 for glutamine).

### Glutamate and Glutamine Levels in the Blood of Patients With Chronic Schizophrenia From Brazil

To validate and extend the above described findings (obtained in cohorts from the USA), we recruited a cohort of patients with chronic schizophrenia from Brazil and compared blood glutamate and glutamine levels with age- and sex-matched healthy controls. Although patient and control groups were quite similar in terms of age and sex, the group of patients with chronic schizophrenia included significantly more smokers than the healthy controls group (*X*^2^ = 13.24, *p* = 0.0001) (Table 4). Thus, we included smoking status as a covariate in the analysis (Table 5). Results showed that patients with chronic schizophrenia had significantly increased blood glutamate levels compared to healthy controls (*F* = 16.3, *p* = 0.0001). Moreover, blood glutamine levels were significantly lower in patients with chronic schizophrenia than in healthy controls (*F* = 55.6, *p* = 0.0001). As a result, the glutamine/glutamate ratio in the blood was significantly lower in patients with chronic schizophrenia than in healthy controls (*F* = 27.0, *p* = 0.0001).

**Table 4:** Characteristics of study subjects with chronic schizophrenia from the Brazilian cohort.

	**Healthy controls**	**Chronic schizophrenia**	***p-*value**
Sex, M/F	34/41	27/40	0.54
Age, years (range)	39.1 ± 13.2 (20–72)	42.4 ± 10.9 (25–68)	0.11
Illness duration, years (range)	N/A	22.4 ± 11.4 (6–54)	N/A
CPZ equivalents (mg/day) (range)	N/A	104.4 ± 94.1 (0–430.2)	N/A
Antipsychotic in use (typical/atypical/combination/no medication)	N/A	22/23/9/1	N/A
Smoker/Non-smoker	7/50	28/39	0.0001
BPRS total score (range)	N/A	42.3 ± 7.7 (24–57)	N/A
BPRS negative score (range)	N/A	16.0 ± 4.9 (6–27)	N/A
BPRS positive score (range)	N/A	25.3 ± 6.3 (10–39)	N/A

*Comparisons between groups were performed using χ^2^ test for sex and smoking status, and t-test for age. Values are shown as means ± standard deviation (range). p-value in bold indicates statistically significant difference. BPRS, Brief Psychiatric Rating Scale; CPZ, chlorpromazine; N/A, not applicable. Typical antipsychotics included flufenazine, clorpromazine, amoxapine, haloperidol, perphenazine, thioridazine, and thiothixene. Atypical antipsychotics included clozapine, olanzapine, quetiapine, risperidone, ziprasidone, and aripiprazol*.

**Table 5:** Blood levels of glutamate and glutamine in chronic schizophrenia (Brazil cohort) using smoking status as covariate.

			**95% C.I**.	
**Amino acids**	**Diagnostic**	**Mean**	**Lower bound**	**Upper bound**	**Statistics**
Glutamate, μmol/L	Healthy Control	264.5	191.8	337.2	*F =* 16.3, *p =* 0.0001
	Chronic schizophrenia	470.0	399.2	540.8
Glutamine, μmol/L	Healthy Control	443.0	404.5	481.4	*F =* 55.6, *p =* 0.0001
	Chronic schizophrenia	242.3	204.8	279.8
Glutamine/ glutamate ratio	Healthy Control	2.21	1.89	2.54	*F =* 27.0, *p =* 0.0001
	Chronic schizophrenia	1.02	0.71	1.34

*C.I., confidence interval*.

**Figure 2:**
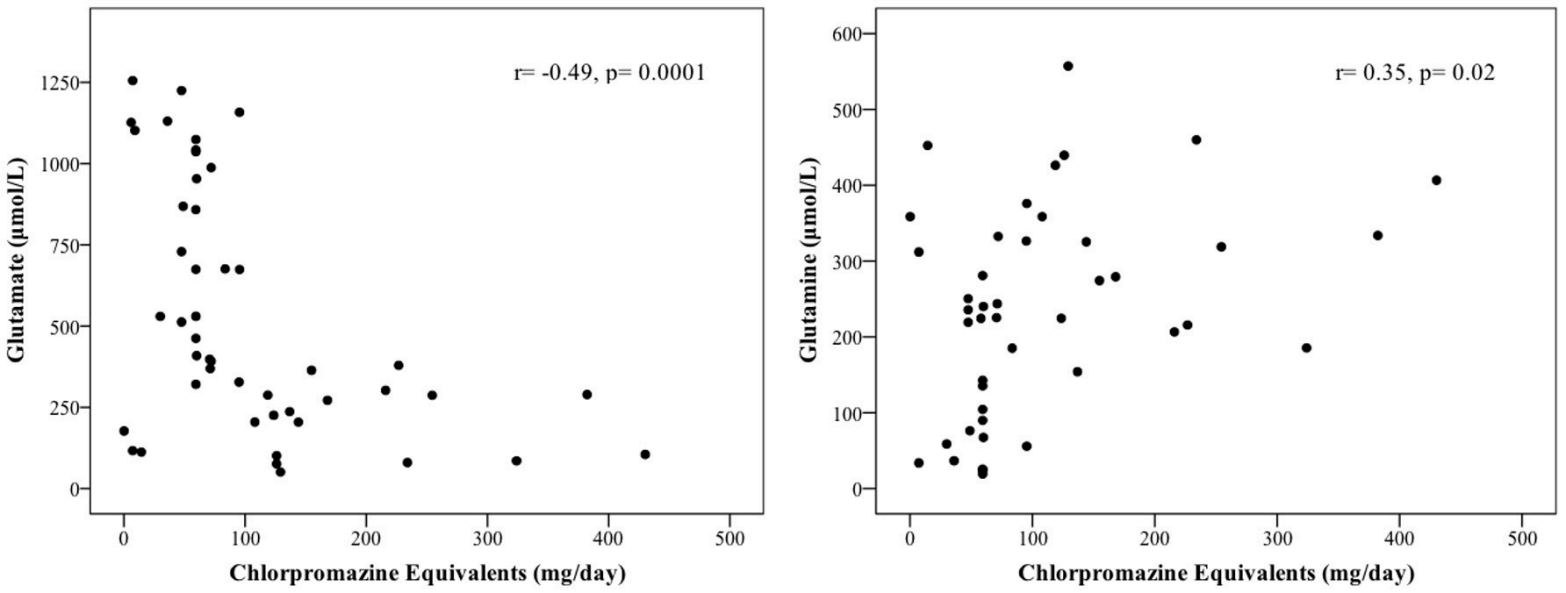
Correlation between blood levels of glutamate and glutamine and the use of antipsychotic medication in chronic schizophrenia (Brazil cohort).

Glutamine levels were negatively correlated to age in healthy controls (*r* = −0.31, *p* = 0.01), but not in chronic schizophrenia patients (*r* = 0.01, *p* = 0.94). Glutamate levels were not correlated to age in both groups (healthy controls: *r* = 0.14, *p* = 0.24; chronic schizophrenia: *r* = 0.01, *p* = 0.92). Glutamate and glutamine levels were significantly correlated to the current use of antipsychotic medications (expressed as chlorpromazine equivalents) (*r* = −0.49, *p* = 0.0001 for glutamate; *r* = 0.35, *p* = 0.02, for glutamine; Figure 2). We compared levels of glutamate and glutamine in patients using typical antipsychotics vs. those using atypical antipsychotics. The glutamine/glutamate ratio was significantly lower in patients using typical antipsychotics than on those using atypical antipsychotics (0.57 vs. 1.25, *F* = 4.23, *p* = 0.046) (Table S1).

We further evaluated whether glutamate and glutamine levels were associated with the severity of symptoms in patients with schizophrenia, measured by their scores on BPRS. Glutamate and glutamine levels were not significantly correlated to total BPRS score (*r* = −0.07, *p* = 0.63 for glutamate; *r* = −0.04, *p* = 0.76 for glutamine), to the BPRS score for negative symptoms (*r* = −0.27, *p* = 0.06 for glutamate; *r* = 0.19, *p* = 0.18 for glutamine), or to the BPRS score for positive symptoms (*r* = 0.03, *p* = 0.84 for glutamate; *r* = −0.15, *p* = 0.31 for glutamine).

## Discussion

The main findings of the present study are that, compared with healthy controls, patients with recent onset schizophrenia showed increased glutamine/glutamate ratio in the blood, while patients with chronic schizophrenia showed decreased glutamine/glutamate ratio. These findings are in agreement with one study that found decreased blood glutamate levels in first episode psychosis (27) and several studies including a meta-analysis that found increased levels of glutamate in the blood in patients with chronic schizophrenia compared with healthy controls (24, 25, 33). The evidence thus indicates that the glutamine/glutamate ratio is increased at the onset of schizophrenia but decreases with the progression of the disorder. Interestingly, the current use of antipsychotic medications (measured in chlorpromazine equivalents) was associated to the levels of glutamate and glutamine in chronic schizophrenia patients. However, the two cohorts showed opposite patterns of correlation: in Brazil more medication was associated to less glutamate and more glutamine, while in the USA patients taking more antipsychotic medications showed more glutamate and less glutamine. It is noteworthy that there were more patients in the Brazilian cohort taking typical antipsychotic (56%) than in the USA cohort (18%). Interestingly we found that patients using typical antipsychotic have lower glutamine/glutamate ratio than patients using atypical antipsychotics, indicating that different antipsychotic may have different effect on glutamate and glutamine levels. Accordingly, studies in patients and in animal models have been showing that some antipsychotic medication affects the release of glutamate in the brain, while others have no effect (37–39).

The increase in glutamate and the decrease in glutamine are in agreement with changes in the enzymes of the glutamate-glutamine cycle previously found in chronic schizophrenia. Studies in post-mortem brain tissue from patients with chronic schizophrenia found decreased levels of the glutamine synthetase protein (19, 20) and increased glutaminase expression and enzymatic activity (18, 21). The consequent decrease in glutamine synthesis and increase in glutamate formation by glutaminase may also explain the significant decrease in glutamine/glutamate ratio observed in patients with chronic schizophrenia compared to healthy controls.

It is important to consider whether the observed changes in glutamate and glutamine in schizophrenia patients could reflect processes occurring peripherally. Although most studies to date have shown positive correlations between blood and cerebrospinal fluid (CSF) levels of glutamate and glutamine (22, 40–43), other studies have reported lack of such correlations (37, 44). Moreover, one cannot exclude the possibility that differences in dietary habits in patients with schizophrenia may account for differences observed here. Patients with schizophrenia often have less healthy diets, consuming less fruit, and vegetables or more calories than the general population (45). Moreover, second-generation antipsychotics may induce altered eating behaviors, including increased susceptibility to hunger and changes in appetite perception (46).

Importantly, studies of glutamate and glutamine levels in the CSF of patients with schizophrenia suggest that our findings in the periphery reflect what is happening in the CNS (41, 43). While patients with chronic schizophrenia showed a significant increase in CSF glutamate levels compared to healthy controls, no difference was observed in patients with recent onset schizophrenia (41). More recently, Hashimoto et al. observed higher glutamine/glutamate ratio in the CSF in patients with recent onset schizophrenia when compared to normal controls (43). Although this argues against a peripheral source for the glutamate and glutamine changes we observed, we have no direct evidence to rule out this possibility.

A limitation of the present study is the absence of a longitudinal follow up of the patients to examine possible changes in glutamate and glutamine levels over time. Moreover, although the differences found in our cross-sectional study were highly significant, the cohorts studied were of modest size and they may not be representative of the population with schizophrenia as a whole.

In conclusion, we found increased glutamine/glutamate ratio in patients with recent onset schizophrenia while in chronic schizophrenia the glutamine/glutamate ratio was decreased, as compared to healthy controls. Given the putative role of dysfunctional glutamatergic neurotransmission in the pathophysiology of schizophrenia, further studies are warranted to elucidate the underlying mechanisms and consequences of the alterations reported here.

## Author Contributions

All authors certify that they have participated sufficiently in the work to take public responsibility for the content. RP and SF participated in the conception and design of study. CM, RP, and SF wrote the manuscript. CM, CV-L, and RP performed the analysis and interpretation of the data. FA, MC, TS, FT, MF, NG, MB, and SV conducted analysis of patients.

### Conflict of Interest Statement

The authors declare that the research was conducted in the absence of any commercial or financial relationships that could be construed as a potential conflict of interest.
