# Supplementary material for: Blood Levels of Glutamate and Glutamine in Recent Onset and Chronic Schizophrenia
Source: Front Psychiatry. 2018 Dec 19;9:713. doi: 10.3389/fpsyt.2018.00713 (PMC6305751; doi:10.3389/fpsyt.2018.00713)

Figure S1: Non significant correlation between age and blood levels of glutamate and glutamine in healthy controls and in recent onset schizophrenia.

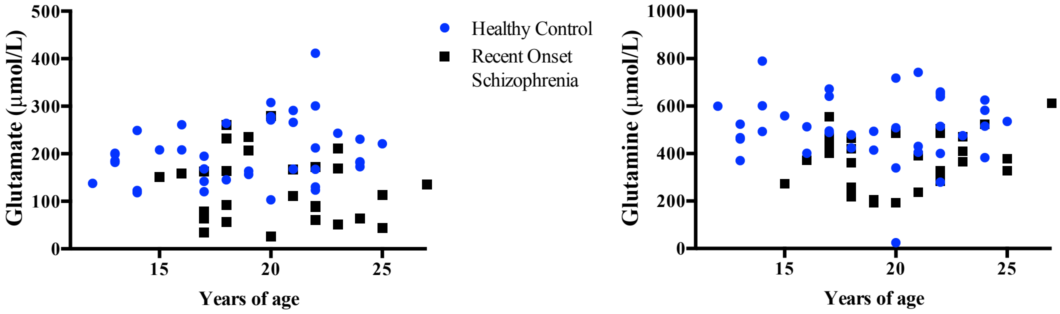

Figure S2: Blood levels of glutamate and glutamine in healthy controls and in recent onset schizophrenia, separated by sex.

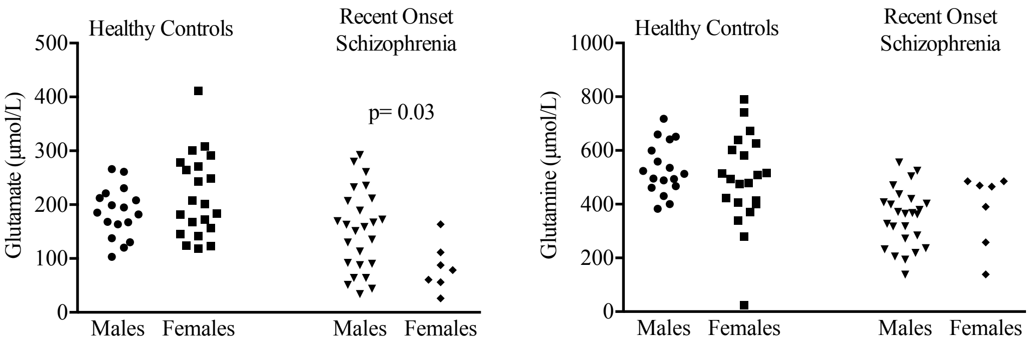

Figure S3: Non significant correlation between blood levels of glutamate and glutamine and use of antipsychotic medication in recent onset schizophrenia.

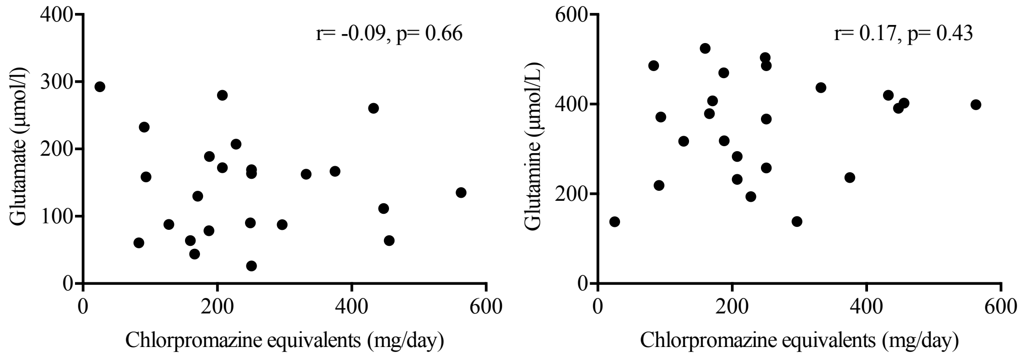

Figure S4: Correlation between age and blood levels of glutamate and glutamine in healthy controls and chronic schizophrenia (USA cohort).

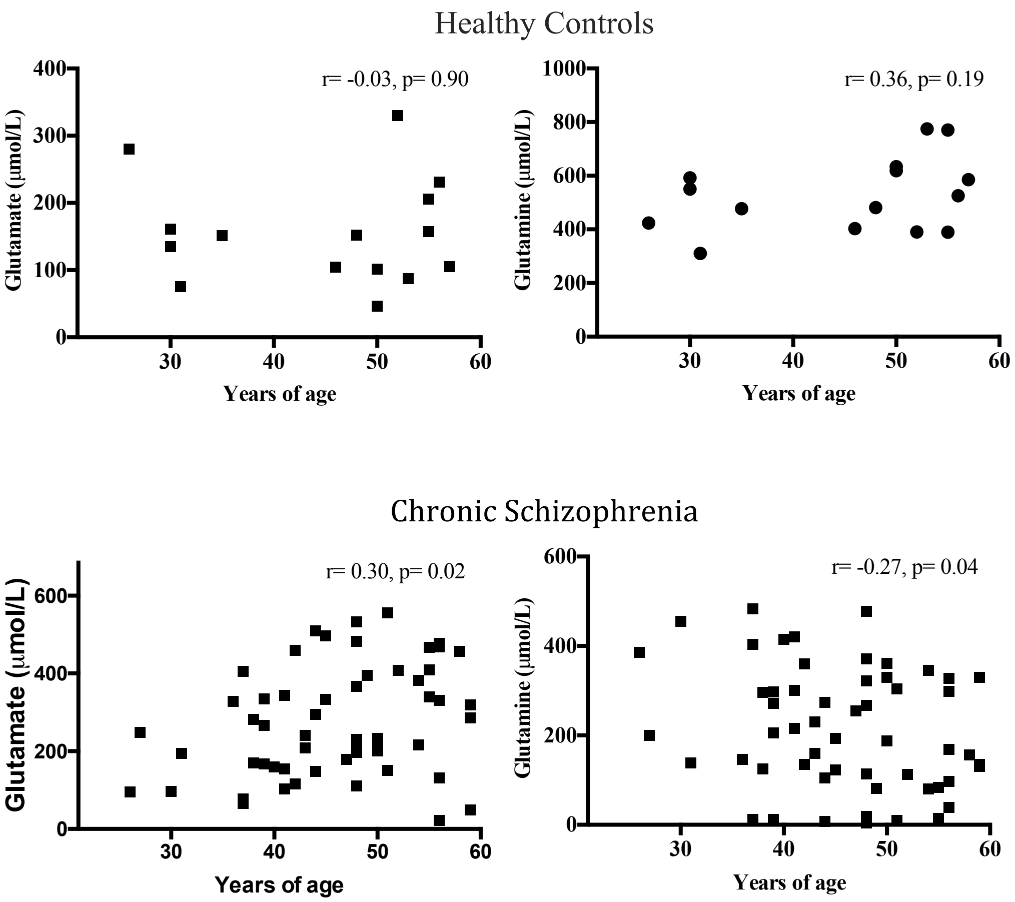

Supplement: Supplementary file 1 [file Data_Sheet_1.PDF]
