# Supplementary material for: Blood Levels of Glutamate and Glutamine in Recent Onset and Chronic Schizophrenia
Source: Front Psychiatry. 2018 Dec 19;9:713. doi: 10.3389/fpsyt.2018.00713 (PMC6305751; doi:10.3389/fpsyt.2018.00713)
Supplement: Supplementary file 2 [file Data_Sheet_2.PDF]

Table S1 - Blood levels of glutamate and glutamine in chronic schizophrenia (Brazil cohort) using smoking status as covariate, comparing patient using typical *versus* atypical antipsychotics.

	Group	Mean	95% C. I. Lower bound	95% C. I. Upper bound	Statistics
Glutamate, μmol/L	Typical	604.8	455.3	754.4	F= 2.07 p= 0.16
	Atypical	449.6	290.8	608.4	
Glutamine, μmol/L	Typical	181.9	122.0	241.8	F= 3.86 p= 0.056
	Atypical	266.8	203.2	330.4	
Glutamine/ glutamate ratio	Typical	0.57	0.12	1.03	F= 4.23 p= 0.046
	Atypical	1.25	0.77	1.73	

C. I., confidence interval.
